# Duplication of the mitochondrial control region is associated with increased longevity in birds

**DOI:** 10.18632/aging.101012

**Published:** 2016-08-11

**Authors:** Ilze Skujina, Robert McMahon, Vasileios Panagiotis E. Lenis, Georgios V. Gkoutos, Matthew Hegarty

**Affiliations:** ^1^ IBERS, Aberystwyth University, Aberystwyth, Wales, SY23 3EE, UK; ^2^ Institute of Cancer and Genomic Sciences, Centre for Computational Biology, Haworth Building, University of Birmingham Edgbaston, Birmingham, B15 2TT, UK; ^3^ Institute of Translational Medicine, University Hospitals Birmingham NHS Foundation Trust, B15 2TT, UK

**Keywords:** control region duplication, mitochondrial genome, birds, lifespan, comparative genomics, genetics, ageing

## Abstract

Despite a number of biochemical and lifestyle differences which should increase risk of oxidative damage to their mitochondrial DNA (mtDNA) and thus reduce expected lifespan, avian species often display longer lifespans than mammals of similar body mass. Recent work in mammalian ageing has demonstrated that functional mitochondrial copy number declines with age. We noted that several bird species display duplication of the control region (CR) of the mtDNA to form a pseudo-control region (YCR), apparently an avian-specific phenomenon. To investigate whether the presence of this duplication may play a similar role in longevity to mitochondrial copy number in mammals, we correlated body mass and longevity in 92 avian families and demonstrate a significant association. Furthermore, outlier analysis demonstrated a significant (p=0.01) difference associated with presence of the YCR duplication in longer-lived avian species. Further research is required to determine if the YCR does indeed alter mitochondrial function or resilience to oxidative damage, but these findings provide an intriguing hint of how mitochondrial sequences may be related to an extended lifespan.

## INTRODUCTION

Mitochondria play an essential dual role in homeotherms by encoding proteins that form the essential components of the mitochondrial energy generation pathway, oxidative phosphorylation (OXPHOS) [1]. OXPHOS generates heat that is used to maintain the organism's body temperature and energy that is utilized for synthesis of adenosine triphosphate (ATP) to perform work [1]. This is achieved “at the cost” of reactive oxygen species (ROS) and free radical production due to electron leakage from the respiratory chain. The mitochondrial theory of ageing suggests that ROS contribute to a progressive accumulation of somatic mutations in DNA during an individual's lifetime leading to both a decline in the bioenergetic function of mitochondria and to cell apoptosis associated ageing [2, 3] and has been associated with a wide range of age-related diseases [1], but the relationship between ROS levels and ageing is not a simple one [27,28]. As an originally free-living prokaryotic organism that was engulfed by a precursor of the modern eukaryotic cell about two billion years ago, cytoplasmic mitochondria have retained their own plasmid-like circular genome [4]. Most of the estimated 2,000 or so original genes have become integrated into the cellular nuclear DNA rendering vertebrate mitochondrial DNAs as circular molecules of around 16.5kb coding for only 37 genes – 13 proteins, 22 tRNAs and a small number of rRNAs [4]. Similar to many bacterial genomes, mtDNA have slightly diverged from the universal genetic code [5, 6] and have hardly any introns within their 15.5 kb coding regions. In vertebrate mtDNA, nearly all the noncoding sequences are concentrated into one single ~1kb long region. This region is commonly referred to as either the *control region* (CR), due to the presence of transcription factor binding sites and mtDNA replication promoter element sequences within it, or the *D-loop region*, as a consequence of its 3D structure during replication. Mitochondrial genome regulation is vital for normal assembly and functional operation of the complexes involved in oxidative phosphorylation (OXPHOS), and therefore, for ATP production and metabolic homeostasis [7]. Besides their main ATP synthesis function, mitochondria are involved in numerous other biosynthetic pathways, such as regulating calcium level stability, intracellular signaling, steroid synthesis, fatty acid metabolism, haem synthesis, the urea cycle, and gluconeogenesis.

Many of these functions are fundamental cellular processes and hence the mitogenome organisation appears highly conserved across vertebrates, and deletions or rearrangements in the mtDNA molecule, up- or down-regulation of mtDNA copy number or point mutations within the mtDNA, can influence the animal's phenotype by promoting selectively (dis)advantageous conditions, cell apoptosis and disease states. In birds, several different arrangements of mitochondrial gene order have been observed and in some species the noncoding CR sequence with the adjacent genes have been duplicated creating a second non-coding region sometimes referred to as the *pseudo control region* (YCR). YCRs appear to have originated independently and sporadically in several distantly related taxa across the avian phylogeny (see, for example, ref. [8]).

Birds have several biochemical and life characteristics that should increase the risk of reactive oxygen species (ROS) damage to their mtDNA relative to mammals. Such characteristics include higher metabolic rates (~1.5–2.5 times higher than similar-sized mammals), higher body temperatures (approximately 3°C higher mean body temperature), higher blood glucose levels (two- to five-fold higher mean blood glucose levels), seasonally high blood lipid levels and very high total lifetime energy expenditures (5 or often 10 times higher [9]). The combined influence of such characteristics should have a negative effect on their longevity according to the mitochondrial theory of ageing. As such one would reasonably predict that relative to mammals, birds should age faster and have higher mutation rates rendering them more prone to cancer and other pathologies. Yet, paradoxically, most birds live longer compared to similar sized mammals in absolute and relative terms [10, 11] and although estimated mutation rates vary greatly across avian phyla (0.0009 - 0.012 s/s/l/Myr), they are on average up to four times lower than those in comparable mammals [12, 13, 14].

Recent work has established that human mitochondrial copy number levels decline with age and that copy number is a heritable trait associated with differences in measurable phenotypes [15, 16]. To the best of our knowledge, the presence of two sets of CR sequences is mainly an avian specific phenomenon, and the relationship between this feature and mitochondrial function has not been fully investigated. Considering the extraordinarily long life spans of birds and the pivotal role of the mitochondria in energy metabolism, the major aim of the work presented here is to explore the possibility that the additional sequences in the YCR are associated with variation in avian longevity.

Around 60% of the variation in lifespan of higher animals can be explained by body mass: animals that diverge from this basic allometry of life span may harbour unique longevity enhancing features and their study may lead to new insights into the evolutionary forces shaping longevity and aging [17]. To explore a possible link between YCR duplication and longevity, we correlated longevity/body mass with presence/absence of YCR sequence for a total of 92 avian families (see Methods).

## RESULTS

**Figure 1:**
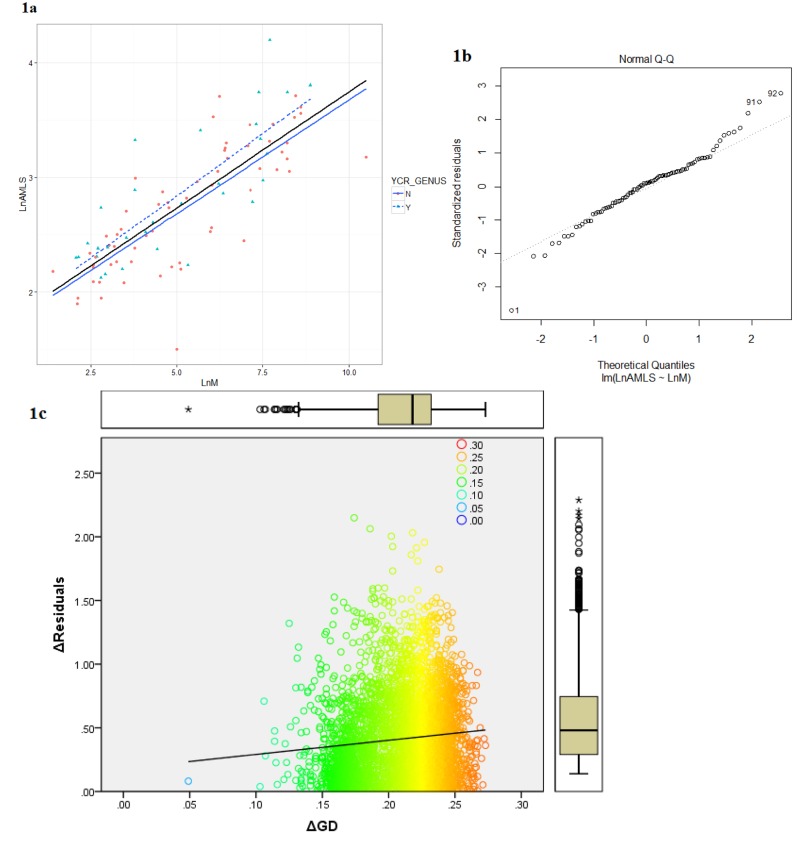
(**a**) The relationship between average family adult body weight (M) and family average maximum life span (AMLS) in Aves: Ln(AMLS) = 0.20Ln(M) + 1.72. (**b**) Quantile-Quantile plot for the linear regression model of the average body mass and longevity for 92 avian families. (**c**) Δresiduals modulus of AMLS and BM as a function of ΔGD (families): y = 1.11x + 0.18, R(**) = 0.009 See Supplement 4 for raw data.

Lifespan and body weight information for 1436 avian species from a total of 149 avian families were determined by combining the datasets of *AnAge* [18], *GlobalSpieces* (http://www.globalspecies.org/) and data collected by Valcu et al. [19] with additional literature searches. The relation of longevity to body mass in 92 bird families selected as having sufficient genomic and life history information from this survey of 1436 species (see Methods) is summarized in Figure 1. On the left (Figure 1a) the natural logarithm of estimated average maximum life span (AMLS) is plotted against the natural logarithm of average recorded adult body weight (LM) and the least squares regression equation for all families combined is given in Table 1. The closeness of the predicted residuals for this distribution with the fitted line are depicted in a Q–Q plot (Figure 1b) – the residual outliers indicated by the divergent curves from the 45° line, depict deviations from a normal distribution in the direction of a positive skew.

**Table 1:** Regression Statistics and Coefficients for lifespan/body mass correlation

Table 1a: Regression Statistics 0	
Multiple R	0.79
R Square	0.63
Adjusted R Square	0.63
Standard Error	0.33
Observations	92

Table 1b Coefficient	Coefficient	Standard Error
Intercept	1.72	0.09
Slope	0.20	0.02

The average body mass was a significant (p<0.05) predictor of the avian family AMLS explaining 60% of the variation (see Table 1) in complete agreement with previous studies based on individual species data [11]. The shape of the Q-Q plot indicates a general fat-tailed Normal distribution of the residuals, with a slight upward skew. Based on the assumption that families that have the largest positive and negative regression residuals are the ones that may have developed evolutionary (dis)advantages leading to prolonged or shortened life histories, we compared the frequency of the pseudo CR duplication between the two groups of families in the extremes of the distribution. In total, 14 “short-lived” (SL) and 10 “long-lived” (LL) families were included in this analysis selected according to the Q-Q plot, using the apparent (though non-significant) discontinuity in the distribution at around +1 and −1 as the cut-off for selecting the representative families. Amongst the SL group, 2 families were pseudo CR positive. In the LL group, 7 out of 10 families were pseudo CR positive. A 2×2 contingency table of these results indicates that pseudo control region sequences are significantly more prevalent in the families with longevity residuals in the upper group than in those families with extremely negative residuals (p=0.01, two tailed Fisher's exact test, Table 2). The regression of delta residual for each family pair against the genetic distance calculated from their coding (*i.e*., non-CR) DNA proved to have a nearly zero slope explaining only a tiny proportion of lifespan differences (r^2^ = 0.009) and with no evidence of an increased size of residuals in most genetically diverse classes (Figure 1c).

**Table 2:** Comparison of YCR presence/absence in long-lived and short-lived avian families

	YCR+	YCR−	Total
**Long-lived**	7	3	10
**Short-lived**	2	12	14
**Total**	9	15	24

Presence/absence of YCR duplication in long and short-lived avian families selected from the extremes of the lifespan/body mass regression. *X^2^* test for difference = 5.531 (Fisher's exact probability, two tailed test, p = 0.01).

Overall, of the 92 families 30 were YCR positive, with 19 of these having positive residuals as opposed to the 62 YCR negative families where half (31) had negative residuals and 31 positive (Supplement 1). Furthermore, for the six families with both pseudo CR positive and negative genera (Supplement 2), we analyzed the ranking of the families with these combined (‘nonsplit’ family containing both YCR positive and negative genera) and separated sub-families (each family ‘split’ into two groups containing either YCR positive or YCR negative species only), with rank 1 being the largest residual below the regression line and 98 the largest residual above the regression (Table 3). Relative ranking was calculated by dividing the absolute rank by the total number of families (n=92 in nonsplit group; n=98 in the split group). Five out of the six positive subgroups were placed above the negative subgroup within their family, suggesting that even within avian families the pseudo control region is associated with longer lifespan. On average, among these paired sub-families the pseudo CR positive species ranked ~17 places higher in the longevity rankings than those with a single CR, 83.83 and 66.67 respectively (Table 3). While these results are strictly non-significant, the analysis shows the same trend as the chi square test: a duplicated control region is more likely to be present in species with above average longevity both within families and across families.

**Table 3:** Ranking of avian families relative to lifespan/body mass regression

Family	Abs. Rank	Rel. Rank	Split group	Abs. Rank	Rel. Rank
Ardeidae	38	0.4368	YCR+	57	0.5816
			YCR−	4	0.0408
Petroicidae	85	0.9081	YCR+	79	0.8061
			YCR−	90	0.9184
Psittaculidae	84	0.8966	YCR+	92	0.9388
			YCR−	85	0.8674
Psittacidae	88	0.9425	YCR+	93	0.9490
			YCR−	88	0.9000
Bucerotidae	87	0.9310	YCR+	96	0.9800
			YCR−	94	0.9592
Turdidae	40	0.4348	YCR+	86	0.8776
			YCR−	39	0.3976

Table 3 shows family position according to its residual value relative to the regression line. Rank 1 corresponds to the shortest lived result according to the predict value (the lowest residual value) and 98 the longest. Relative rank is calculated dividing the absolute position by the total number of families.

## DISCUSSION

Although avian species have been used as model organisms in numerous biogerontology studies, the emphasis has been largely restricted to physiological aspects of bird longevity, such as cell membrane fatty acid composition [11], telomere length dynamics, oxidative stress management, hormonal and immune function [20]. We have detected a novel relationship between the presence of a genetic feature of the mitochondria, a duplicated control region, and longevity. The mechanism underlying this relationship requires further investigation. In a recent phylogenetic study of mtDNA CR region duplications in parrots [8], the hypothesized multiple duplications are associated with subsequent differential loss of flanking sequences (extra copies of tRNAs and coding genes) in different species but maintenance and even co-evolution of primary and duplicated CR domain sequences within the duplicated mitochondria.

This, we believe, strongly argues for a positive functional role of these duplicated sequences in those species that carry them. We have demonstrated here a positive relationship between such duplications and longevity across a broad spectrum of the avian phylogeny and hypothesize that there are two, not incompatible, possibilities that relate to the mito-chondrial ageing hypothesis. Extra control region sequences may result in constant increased mtDNA copy number and/or increased flexibility and speed of cellular response when increased metabolism is required to cope with environmental stresses. This mechanism might effectively lower local ROS damage from increased metabolic throughput during periods of stress response. A second possibility is that extra copies of the control region sequences protect mtDNA from the age related effects of sequence losses and hence offer the opportunity for species to retain higher levels of functional mitochondria into later life, hence slowing the negative effects of accumulated mitochondrial deletions on senescence. Alternatively our results may simply reflect a molecular marker of long-lived species, rather than being the causative agent for that longevity. This would in part explain why our results are less significant for the split data since we would be partitioning ‘long lived genera’ into YCR+ and YCR− species (hence reducing the number of relevant data points being tested) rather than looking at independent long lived species versus short lived species as the statistical test assumes. Our results suggest that even if truly causative the associated effect is a relative minor one accounting for around a 15% difference in life expectancy for an average sized family with and without the YCR. As mentioned in the methods, the relatively patchy distribution of both molecular and phenotypic data across the whole bird phylogeny greatly reduces the statistical power of our assay to determine the extent of this small effect. In particular differential levels of study of particular taxa will have been likely to bias longevity estimates in a downwards direction, and we identified several such potential examples while reviewing the databases. For example, the House Sparrow (*Passer domesticus*) is a very widely studied small species (25g) with an “Acceptable” data quality and “Medium sample size” in the *AnAge* database and 2729 records in Valcu's et al. [19] database originating from thousands of banding studies. This species is reported to live up to 23 years: whereas the recorded MLS from low number of banding studies (53 records in Valcu et al. (2014) and “Small sample size” with “Not yet established” longevity in *AnAge*) for the closely related passerine *Ammodramus bairdii* (Baird's Sparrow) weighing a similar 19g, is a mere 4.6 years. This could reflect a true 5 fold difference in MLS between these two passerine families, but is more likely an artefact of observation – the failure to observe a 23 year old Baird's Sparrow with minimal records is not the same as proof that Baird's Sparrows cannot live as long as House Sparrows.

Although small, a 15% difference in the onset of pathologies associated with human senescence would represent a significant saving in health care costs and a reduction in morbidity if it could be achieved. Both of our suggested routes by which an extra CR could contribute to mitochondrial function and ageing raise the possibility of manipulation of the senescence process through direct manipulation of mitochondrial function. However, we have not excluded the possibility that these observations are indicative of other mechanisms acting in birds and further research is certainly required to understand the relationship between this mitochondrial feature and lifespan.

## CONCLUSION

Bird species live longer on average than mammals of a similar size and many species have been found to harbour independently arisen rearrangements of their mitochondria that include duplications of the mitochondrial Control Region sequences. In this research we have established, albeit for a restricted number of avian families, a significant association between the presence of these additional mitochondrial CR sequences and longevity across a wide phylogenetic range of birds. We propose two mechanisms by which such duplications could increase the life span of a species, either by protecting cells from loss of mitochondrial function from age related deletions, or through increased potential flexibility of mitochondrial response to environmental changes. Although our results are preliminary, we believe they indicate a novel target for further investigation of the relationship between mitochondrial function and senescence in birds and other vertebrates.

## METHODS

Lifespan and body weight information for 1436 avian species were determined by combining the datasets of *AnAge* [18], *GlobalSpieces* (http://www.globalspecies.org) and Valcu et al. [19] as well as literature information. In ambiguous cases, where the datasets had different records for a species, the longest maximum life span (MLS) was used. Using MLS as a potential lifespan measure poses at least four major problems:

- the records are not uniform as a result from originating in various different ways depending on a record source (*i.e*., ringing or zoo records);
- MLS is not an ideal measure of ageing as observed life spans can be affected by factors other than natural senescence [21, 22];
- errors are most likely to be biased by the degree of study of a species in that they will tend to consistently underestimate MLS in poorly studied species.
- at the other extreme, each record may represent a single life event for a single individual animal, therefore it may be under- or over-representative of the general trend for that species or group.

It should also be borne in mind that many life-history traits that may significantly affect life expectancy, such as the distribution of recorded lifespans observed or environment (or range of environments) in which records are obtained are not routinely available for every species. A search of NCBI identified those records with either complete mtDNA or sufficient mtDNA sequences to determine the presence of a complete or partial duplication of the CR sequences in 517 birds (as of June, 2016). Of these 517 species only 216 had sufficient records in our lifespan and bodyweight database to be used in this study, with the distribution of information being unequal across the avian phylogeny.

In order to minimize the impact of these problems, we examined the avian body mass and lifespan by pooling and averaging data within families where life history and genetic information were present for more than one individual species per family. Following this approach, the stochastic and unusual events, *i.e*., a single value for a single individual – were omitted leaving trimmed mean values for each family. This resulted in a drastic reduction in the number of observations available, and thus the statistical power (from n=1436 individuals to n=92 families), but is likely to minimize bias by representing the central tendency of each group of species, smoothing out the bias associated with underestimation of maximum lifespan in poorly studies species.

In total, 149 avian families were represented from our original dataset: 41 of these were excluded as phenotype data was available for less than 3 genera of the same family (the exceptions being *Anseranatidae, Balaenicipitidae, Pandionidae, Sagittariidae, Psittaculidae-pseudo CR positive group, Bucorvidae, Turdidae - pseudo CR negative group* which consist of only a single broad genus). A further 16 families were excluded due to the lack of either complete mitochondrial sequence or maximum lifespan information, leaving a total of 92 avian families with life history and sequence information (Supplement 3).

The complete mtDNA genomes were accessed and downloaded from NCBI (http://www.ncbi.nlm.nih.gov/ nucleotide, 25/06/2016) as FASTA files by initially querying the database for all ‘complete’ mitochondrial databases then searching for each individual genus to ensure that a consensus reference genome was used if available. The complete mtDNA coding sequences, excluding the CR(s), were then aligned with Clustal Omega [23] by using the default parameters and the estimates of genetic distance for all the pair-wise comparisons obtained according to MEGA 7 [24] by using the Maximum Composite Likelihood model with Gamma distributed (G) rates among sites [25]. The total genetic distances between all family pairs were then calculated and correlated with the estimated difference in MLS to test the hypothesis that longevity differences were related to genetic changes in the coding genes of the mitochondria.

The downloaded sequences were then manually checked for YCR presence and other mitochondrial sequence rearrangements as described in [26]. The whole family was labelled as YCR positive or negative, if there were at least one species present with a complete mitochondrial genome sequence that had annotated CR and/or YCR or the presence/absence of the YCR could be deducted from the typical gene order. The accession numbers of the sequences used are listed in Supplement 2. Six families – *Ardeidae*, *Petroicidae*, *Psittaculidae*, *Psittacidae, Turdidae* and *Bucerotidae* contained individual genera both with and without the pseudo control region within the family. In these cases, the family was treated as pseudo CR positive family in the initial analysis (Supplement 3, other structural rearrangements are indicated by letters corresponding to the gene orders summarized in [26]), the regression analysis was subsequently also performed by separating these 6 families into 12 sub-families, 6 containing only pseudo CR positive species and 6 only pseudo CR negative species *(e.g. Ardeidae_Y & Ardeidae_N etc)*[ Supplement 2].

A liner regression model was created in R and used to calculate the residual values for each data point using natural logarithm transformed average body mass (in grams) and longevity (in years) for the 92 families. The outlying regression residuals were examined by quantile-quantile probability plot produced in R. In order to check for biased covariance of phylogenetic distance and longevity, the correlation between genetic distance between each family pair and the difference in residuals between them was calculated and is shown in Figure 1c.

## SUPPLEMENTAL DATA FIGURE AND TABLE


