# Supplementary material for: Duplication of the mitochondrial control region is associated with increased longevity in birds
Source: Aging (Albany NY). 2016 Aug 11;8(8):1781–8. doi: 10.18632/aging.101012 (PMC5032695; doi:10.18632/aging.101012)
Supplement: Supplementary file 1 [file aging-08-1781-s001.pdf]

SUPPLEMENTAL DATA

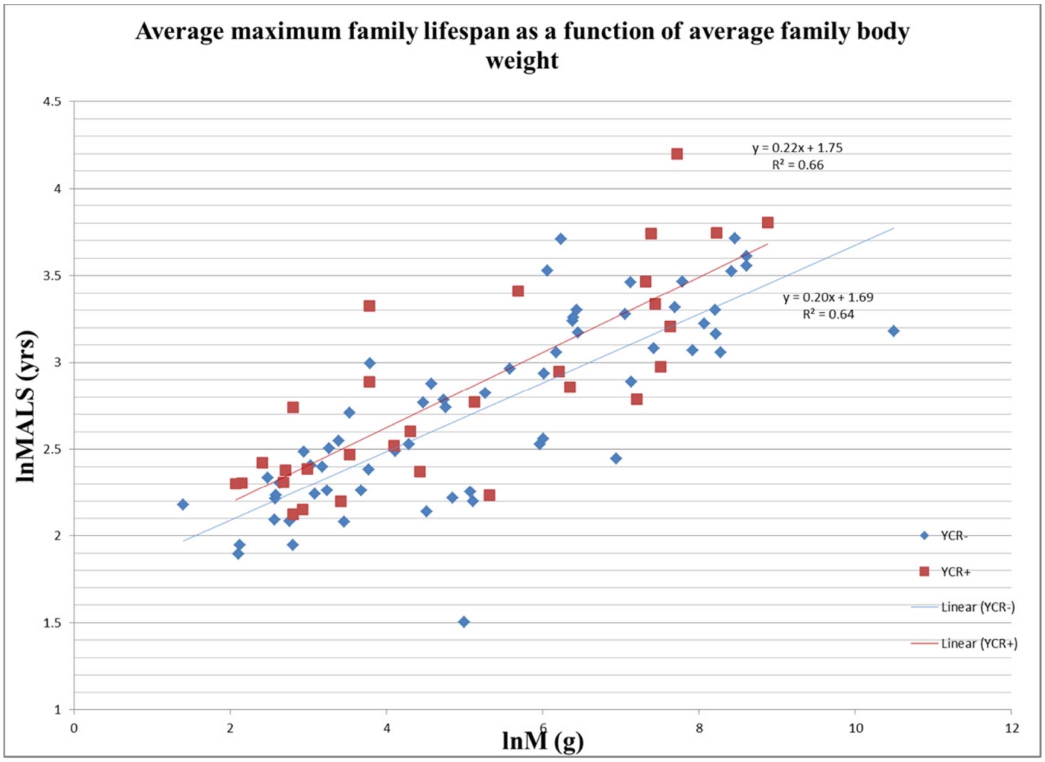

Supplemental Figure 1. Distribution of average YCR+/- family lifespan against average body weight.

Supplemental Set 1. Binomial distribution table.

Probability of success on a single trial = 0.5	YCR+	YCR-
No of trials	30	62
No above the line	19	31
P-value	0.05	0.10

http://stattrek.com/online-calculator/binomial.aspx

Please browse Full Text version of this manuscript to see Supplemental materials:
Supplemental Set 2. List of raw data for all families split by YCR status
Supplemental Set 3. Raw data for regression and QQ plot - mixed families treated as single groups.
Supplemental Set 4. raw data for the correlation of genetic distance between species with difference in life expectancy relative to expectation from body weight regression
